# P45 Forms a Complex with FADD and Promotes Neuronal Cell Survival Following Spinal Cord Injury

**DOI:** 10.1371/journal.pone.0069286

**Published:** 2013-07-23

**Authors:** Tsung-Chang Sung, Zhijiang Chen, Sandrine Thuret, Marçal Vilar, Fred H. Gage, Roland Riek, Kuo-Fen Lee

**Affiliations:** 1 Clayton Foundation Laboratories for Peptide Biology, The Salk Institute, La Jolla, California, United States of America; 2 Centre for the Cellular Basis of Behaviour & Medical Research Council Centre for Neurodegeneration Research, Institute of Psychiatry, King’s College London, London, United Kingdom; 3 Neurodegeneration Unit, Instituto de Salud Carlos III, Madrid, Spain; 4 Laboratory for Physical Chemistry, Eidgenössische Technische Hochschule Zürich, Zürich, Switzerland; Inserm, France

## Abstract

Fas-associated death domain (DD) adaptor (FADD), a member of the DD superfamily, contains both a DD and a death effector domain (DED) that are important in mediating FAS ligand-induced apoptotic signaling. P45 is a unique member of the DD superfamily in that it has a domain with sequence and structural characteristics of both DD and DED. We show that p45 forms a complex with FADD and diminishes Fas-FADD mediated death signaling. The DED of FADD is required for the complex formation with p45. Following spinal cord injury, transgenic mice over-expressing p45 exhibit increased neuronal survival, decreased retraction of corticospinal tract fibers and improved functional recovery. Understanding p45-mediated cellular and molecular mechanisms may provide insights into facilitating nerve regeneration in humans.

## Introduction

The death domain (DD) superfamily consists of proteins that have a characteristic DD fold or DD-like domains. This superfamily can be divided into four subfamilies based on the structural and amino acid differences in the DD and DD-like domains: the DD subfamily, the death effector domain (DED) subfamily, the caspase recruitment domain (CARD) subfamily and the pyrin domain (PYD) subfamily [1]. These domains serve to mediate interactions among members of the DD superfamily to initiate signaling cascades. Some members of this superfamily contain multiple domains. For example, Fas-associated death domain (FADD) protein, an adaptor molecule for Fas signaling, contains both a DD and a DED. Caspase-8 contains a DED and an enzymatic activity domain. Following Fas Ligand (FasL) engagement, FADD is recruited to bind Fas (CD95/Apo-1) through a DD-DD interaction. The DED on the FADD then recruits the caspase-8 through a DED-DED interaction, leading to the formation of the death-inducing signaling complex (DISC).

The DD superfamily is best known for its role in eliciting apoptotic signaling cascades [1], but several lines of evidence have demonstrated that DD members also play essential, non-apoptotic roles in diverse biological systems, including the nervous system [2], [3]. These non-apoptotic activities include survival, nerve growth, neuroplasticity and nerve regeneration. For example, the role of the p75 neurotrophin receptor (p75) and Fas has been intensively studied in the nervous system during development and in degeneration and regeneration [4]. Depending on the cellular context, both Fas and p75 can elicit signals that either promote or prevent neuronal survival and nerve growth. For example, FasL acting through Fas is capable of simultaneously eliciting apoptotic signaling via the activation of caspase-8 and anti-apoptotic signaling via the activation of NF-κB and Erk1/2 [5]. Fas-mediated cell death plays a role in nerve degeneration of the central nervous system [6], [7]. For example, levels of Fas and FADD are elevated and are correlated with increased cell death following spinal cord injury [8], [9], [10], [11], [12].

A 45 kD transmembrane (TM) glycoprotein (p45) that bears a high degree of homology to the p75 neurotrophin receptor has been identified by others and by our group and is also called PLAIDD (p75-like apoptosis-inducing death domain protein) [13], NRADD (neurotrophin receptor alike DD protein) [14] or NRH2 (neurotrophin receptor homolog 2) [15]. P45 increases nerve growth factor (NGF) binding affinity to TrkA and enhances NGF signaling [16], [17]. Recently, Kim *et al.* showed that p45 is a trafficking switch to regulate sortilin localization [18]. Here we show that p45 forms a complex with the FADD adaptor. P45 attenuates FasL-induced caspase activation. Consistent with these findings, p45 markedly inhibits FasL-induced cell death. Transgenic mice over-expressing p45 exhibit a significant increase in neuronal survival and functional recovery in response to nerve injury. These results establish a novel neuroprotective role for p45 after nerve injury.

## Materials and Methods

### Cell cultures

HEK293 cells were cultured in the DMEM medium supplemented with 10% fetal calf serum, 100 units/ml penicillin and 100 µg/ml streptomycin. MCF7 cells (a human breast adenocarcinoma cell line) were grown in the RPMI medium supplemented with 10% fetal calf serum, 0.01 mg/ml insulin, 100 units/ml penicillin and 100 µg/ml streptomycin.

### Transfection and Immunoprecipitation

The cDNAs of mouse WT FADD and a variety of point-mutated FADD created by the QuikChange site-directed mutagenesis kit (Stratagene) were V5-tagged and cloned into pcDNA3.1 (+) vector. Constructs were transfected into a stable HEK293 cell line expressing CrmA and Flag-p45 by TransFectin™ reagent (Bio-Rad). Transfected cells were collected and lysed in RIPA buffer (150 mM NaCl, 1% NP-40, 0.5% DOC, 0.1% SDS, 50 mM Tris pH 8.0). Lysates were immunoprecipitated with an anti-Flag M2 antibody (Invitrogen). Samples were analysed using SDS-PAGE and Western blots. For the analysis of DISC formation, cell extracts were immunoprecipitated with an anti-FAS antibody and immunoblotted with an anti-caspase-8 antibody. For the analysis of p45 and FADD interaction in the nervous system, brain or spinal cord extracts were immunoprecipitated with an antibody against p45 or FADD and immunoblotted with an antibody against FADD or p45, respectively. Antibodies against Fas, FADD and caspase-8 were purchased from Santa Cruz Biotechnology.

### Generation of Thy1-p45 Transgenic Mice

Thy1-p45 transgenic mice were generated using the standard protocol. Briefly, a full-length mouse p45 cDNA containing a Flag tag at the N-terminal was cloned into the Xho I site of the Thy1 expression cassette. The transgene construct was injected into one-cell embryos of DBA X C57/B6.

### Spinal Cord Injury (SCI)

Using standard aseptic techniques, 12-week-old littermates of female controls and Thy1-p45 transgenic mice (10 each) were anesthetized with ketamine (100 mg/kg intraperitoneally) and xylazine (10 mg/kg intraperitoneally) and received a dorsal laminectomy at T9. The spinal cord was exposed and 1% lidocaine was applied to the dura for 1 minute to anesthetize the region to be transected. The entire depth of the spinal cord was transected with a sterile ophthalmic scalpel (15 degree angle). Durafilm was inserted over the laminectomy site to prevent tissue adherence to the spinal surface. The overlaying muscle layers were sutured (absorbable surgical suture (Ethicon®, chronmic gut 4-0)) and the skin was stapled close. Postoperatively, all animals were allowed to recover on warming pads with moist food pellets on the bottom of the cage. Temperature and respiration were monitored until the animals awakened. When the mice were alert, the cages were placed back on the housing system rack. Bladders were manually expressed twice a day. Antibiotic (enrofloxacin, 2.27 mg/kg; Baytril) and analgesia (buprenorphine 0.01 mg/kg) were given daily for 7 days. Animals were anesthetized and transcardially perfused with saline, followed by 4% paraformaldehyde at 6 weeks post-SCI. Animal protocols were approved by the Institutional Animal Care and Use Committee (IACUC) of the Salk Institute and The Council on Accreditation of the Association for Assessment and Accreditation of Laboratory Animal Care (AAALAC International).

### Stab Wound Injury

The standard surgical procedures of SCI as described above were followed. Instead of a complete transection, the spinal cord was stabbed with a sterile ophthalmic scalpel (15 degree angle) on the right side of T13 spinal cord as described previously [19].

### TUNEL Assay

Spinal cord cryo-sections (20 µm) were used for TUNEL assay following the protocol as described in the manual of “In Situ Cell Death Detection Kit, TMR red” (Roche).

### Locomotor Behaviour Analysis

Open field observations of locomotor behaviour were scored using a new locomotor rating scale, the Basso Mouse Scale (BMS), which was developed specifically for mice [20]. The BMS scale is based on a systematic analysis of locomotor recovery from SCI in mice. The characteristics of locomotor recovery are different in mice than rats, and the numeric ranking system of the new scale takes these differences into account. The BMS scale also includes a subscore to further differentiate between animals that are plantar stepping by awarding points for plantar stepping, coordination, paw position, trunk stability and tail position. The scale is from 1 to 9. Use of the BMS for locomotion in mice has been shown to be a more sensitive and reliable indicator of recovery than the BBB scale [21]. The surgery and behavioural analyses were performed in a double-blind manner.

### Histological Analyses

Thy1-p45 transgenic mice and their WT littermates received SCI, and CST fibers were labeled by either axon tracing with BDA injected into the sensorimotor cortex or genetic YFP labeling by mating Thy1-p45 transgenic mice and their WT littermates with Emx1-Cre/Thy1-STOP-YFP mice. For anterograde BDA labeling of the CST fibers, spinal cord-injured mice received CST tracing at 28 days post-injury. Mice were anesthetized as described above and a hole was drilled on each side of the skull overlying the sensorimotor cortex. The anterograde neuronal tracer, BDA (10% BDA in 0.01 M phosphate buffer; Molecular Probes, Eugene, OR), was injected (2 µl) at 4 injection sites on each side into the sensorimotor area using a Hamilton syringe. Two weeks after the BDA injection, these animals were perfused and tissue was collected for histology. The spinal cords were sliced sagittally using a cryostat at 20 µm thickness. The sections were incubated with avidin–biotin–peroxidase complex, and the BDA tracer was visualized by nickel-enhanced diaminobenzidine HRP reaction. For genetic YFP labeling, the spinal cord samples were collected after 6 weeks of SCI and sectioned sagittally using a cryostat at 20 µm thickness. YFP fluorescence was readily visible under a fluorescence microscope. To detect fine axon fibers, YFP signal was amplified with an anti-GFP antibody (1∶1,000, Rockland) using the standard Nickel-DAB immuno-staining protocol.

### CST Fiber Retraction Analysis

Three qualified sections from each mouse were analyzed. There was clear formation of a scar at the injury site marked by the invasions of other cells (for example, glial cells, Schwann cells and fibroblasts etc.) and discontinuity of white and grey matters after 6-week of SCI. The lesion epicenter was defined as the middle point of the scar on each sagittal spinal cord section. Similar to the CST fiber retraction analysis described before [22], the distance between the lesion epicenter and the main CST fiber front was measured. The main CST fiber front was defined as the point closest to the lesion epicenter where adjacent fibers form a fascicle of approximately 100 µm wide or more.

### Statistical Analyses

All the behavioural data and quantification of traced CST fibers were analyzed using a two-factor ANOVA. Post hoc analysis was carried out using Bonferroni-corrected individual comparisons (Microsoft Excel and XLSTAT; Addinsoft, NY). Statistical evaluation and significance of the other data were determined by two-tailed Student’s t-test. In all analyses, a P-value of less than 0.05 was chosen as significance threshold. All data are presented as means ± standard error of the mean (s.e.m.).

## Results

### The DED of FADD is Required for the Complex Formation with p45

**Figure 1:**
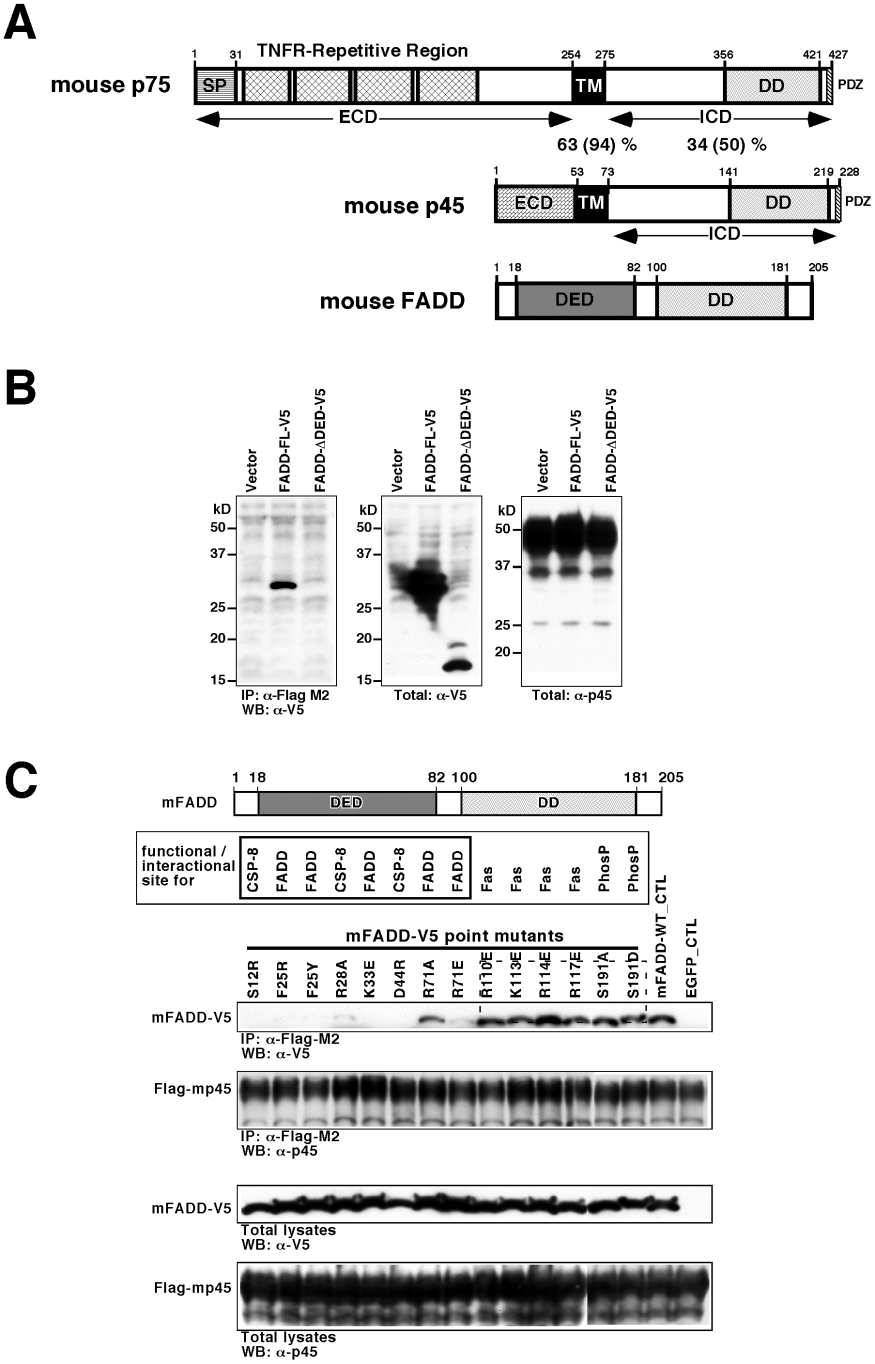
P45 forms a complex with FADD. (A) Schematic representation of the domains in the mouse p75, p45 and FADD (SP, signal peptide; TNFR, tumor necrosis factor receptor; ECD, extracellular domain; TM, transmembrane domain; ICD, intracellular domain; DD, death domain, PDZ, post synaptic density protein, Drosophila disc large tumor suppressor and zonula occludens-1 protein domain; DED, death effector domain) (B) Full-length FADD is essential for the p45-FADD interaction. V5-tagged full length FADD, FADD-ΔDED or FADD-ΔDD (data not shown) was co-transfected with Flag-tagged p45 into HEK293 cells. The lysates were immunorecipitated by an anti-Flag M2 antibody and immunoblotted with an anti-V5 antibody. (C) Both FADD dimerization and FADD-Caspase-8 (CSP-8) interaction are critical for p45-FADD interaction. A series of FADD point mutants were created according to reports in the literature. Schematic representation of the domains in FADD is shown (DED, death-effector domain; DD, death domain). Point-mutated amino acid in FADD is indicated above for its protein-binding ability or individual function (CSP-8, caspase-8/Flice; PhosP, phosphorylation sites). Interaction between FADD and FAS and the serine phosphorylations of FADD are not essential for p45-FADD interaction. In contrast, the binding sites for FADD dimerization and FADD-Caspase-8 interaction, which are located in the α3 helix in the FADD death effector domain, are critical for p45-FADD interaction, suggesting that FADD dimerization or FADD-Caspase-8 interaction might be essential for sequential p45-FADD interaction. (C, D).

P45 is a homolog of p75. They share a high degree of amino acid similarities in their TM (94%) and intracellular domains (ICD) (50%), including the DD (Figure 1A). In contrast, the p45 extracellular domain (ECD) is short and divergent in sequence compared to the p75 ECD and, hence, lacks the binding domain for neurotrophins. Nuclear magnetic resonance (NMR) studies show that p45 ICD contains a flexible domain at the N-terminus (residues 75–140) and a folded domain at the C-terminus (141–219). As seen in other DD members, the 3-dimensional NMR structure of the folded p45 DD contains six α-helices. Interestingly, a RxDφ motif (x, any residue; φ, hydrophobic residue), which is typically observed in DEDs such as those from PEA-15, FADD, Caspase-8 and others [23], [24], is found at the beginning of helix α6, suggesting that the p45 DD has sequence and structural characteristics of both DD and DED (Vilar *et al*., Submitted) and may play a unique role in signaling. Expression studies show that p45 and p75 are co-expressed in numerous tissues, including the brain, spinal cord, dorsal root and sympathetic ganglia. Expression levels are high in embryonic tissues but are dramatically decreased in the adult (data not shown).

As DD and DED serve to mediate interactions among members of the DD superfamily to initiate signaling cascades, we studied whether p45 forms a complex with members of the DD superfamily, including TNFR1, Fas, p75 and FADD. As shown in Figure 1B, p45 forms a complex with FADD when co-transfected into HEK293 cells, but not with TNFR1, Fas or caspase-8 (data not shown). We then set out to determine whether DD or DED of FADD was required for the interaction with p45. As shown in Figure 1B, deletion of DED abolished the complex formation between FADD and p45. It appears that FADD without the DD is unstable (data not shown). Thus, we could not assess the importance of its DD in complex formation with p45 using deletion mutants. Therefore, we generated point mutations of amino acids in the DED or DD of FADD that were important for FADD to interact with itself, with caspase-8, or Fas [24], [25], [26], [27], [28], [29]. The results showed that all amino acid mutations within the DED of FADD that abolish the interaction between FADD and caspase-8 or FADD itself are able to abolish its complex formation with p45 as well, whereas mutations within the DD of FADD that abolish its interaction with Fas had no effect on its complex formation with p45 (Figure 1C). These results suggest that DED of FADD is essential for the p45-FADD interaction; however, the contribution from DD of FADD to the interaction between p45 and FADD cannot be completely ruled out.

### P45 Attenuates FasL-induced Caspase-8 Activation and Cell Death

**Figure 2:**
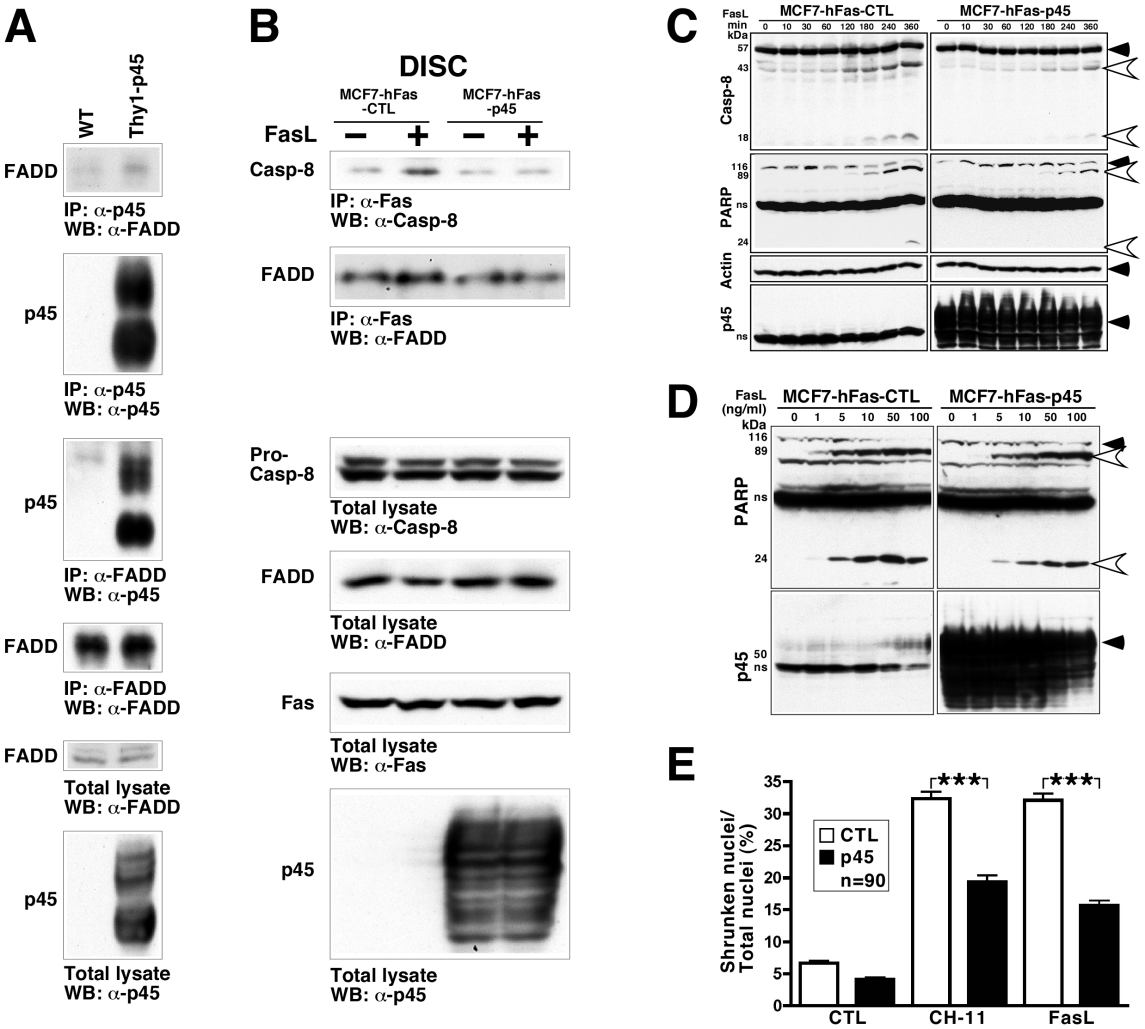
P45 reduces FasL-induced cell death signalling. A. Brain protein extracts from controls and the Thy1-p45 transgenic mice were immunoprecipitated with an anti-p45 or an anti-FADD antibody and followed by immunoblotting with an anti-FADD or an anti-p45 antibody, respectively. The efficiency of immunoprecipitation and expression levels of p45 and FADD were validated with appropriate antibodies as indicated. B. MCF7-hFAS-CTL and MCF7-hFAS-p45 cells were treated with FasL (10 ng/ml) for 0 (–) or 30 (+) min. The lysates were immunoprecipitated with an anti-Fas antibody and followed by immunoblotting with an anti-caspase-8 (Casp-8) antibody. The recruitment of Casp-8 to the DISC is markedly reduced by p45, but not FADD. The efficiency of immunoprecipitation and expression levels of Pro-casp-8, FADD, Fas and p45 were validated with appropriate antibodies as indicated. (C, D) MCF7-hFAS-CTL and MCF7-hFAS-p45 cells were treated with FasL (10 ng/ml) for 0–360 min. The lysates were immunoblotted with an anti-caspase-8 or an anti-PARP antibody. The cleavage of both proteins is markedly attenuated in p45-expressing cells. An anti-actin antibody was used for protein loading control. Closed arrowhead, full-length protein; open arrowhead, cleaved products. (E) FasL- or anti-Fas antibody CH11-treated cells were stained with DAPI to detect shrunken nuclei, indicative of cell death. P45-expressing cells significantly reduced cell death induced by either treatment. ***P<0.001. Data are represented as means ± s.e.m.

**Figure 3:**
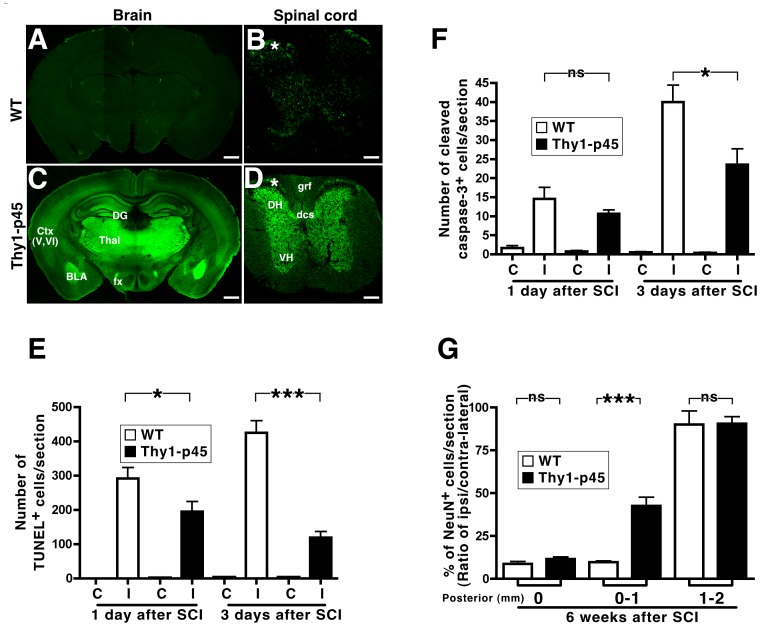
Thy1-p45 transgenic mice display decreased cell death following stab wound injury of the spinal cord. (A–D) Brain (A, C) and spinal cord (B, D) sections from WT littermates (A, B) and Thy1-p45 transgenic mice (C, D) were immunostained with an anti-p45 antibody. Ctx (V, VI), cortex layers V&VI; BLA, basolateral amygdala; DG, dentate gyrus; Thal, thalamus; fx, fibers of fornix; DH, dorsal horn; VH, ventral horn; grf, gracile fasiculus; dcs, dorsal corticospinal; *, spinal projection fibers from dorsal root ganglia neurons. Scale bar, 1 mm (A, C); 200 µm (B, D). (E–F) WT and Thy1-p45 mice were subjected to stab wound injury in the right side of the spinal cord at the T13 level. One and 3 days after injury, sections were stained for TUNEL^+^ (E) and activated caspase-3^+^ (F) cells. The numbers of TUNEL^+^ and activated caspase-3^+^ cells were counted in the half spinal cord contralateral (C) and ipsilateral (I) to the stab wound injury. The numbers of TUNEL^+^ and activated caspase-3^+^ cells in the ipsilateral side were markedly lower in the Thy1-p45 mice. (G) Six weeks after stab wound injury, the number of NeuN^+^ cells within 1 mm from injury site was significantly higher in the Thy1-p45 mice compared to their WT littermates, suggesting that p45 expression may decrease neuronal cell death. *P<0.01. ***P<0.0001. ns, not significant. Data are represented as means ± s.e.m.

We investigated whether p45 and FADD form a complex *in vivo* (Figure 2A). Because p45 expression in the nervous system is markedly reduced in the adult, we take advantage of transgenic mice over-expressing p45 (see Figure 3A for description of the transgenic mice). Protein extracts were prepared from the brain, immunoprecipitated by an anti-45 or an anti-FADD antibody and followed by immunoblotting with an anti-FADD or an anti-p45 antibody, respectively. The results showed complex formation between p45 and FADD (Figure 2A). Similar results were obtained when spinal cord extracts were used (data not shown).

Following the aggregation of Fas and FADD, caspase-8 is recruited to the death inducing signaling complex (DISC) and activated by self-cleavage. The activated caspase-8 triggers a step-wise cleavage and activation of a chain of effectors for executing cell death, including caspases-3 and -7. These executor caspases function via cleaving a variety of substrates, including Poly (ADP-ribose) polymerase-1 (PARP-1), which when activated, protects the genome by functioning in the DNA damage surveillance network. PARP-1 activation is also required for the translocation of apoptosis-inducing factor (AIF) from the mitochondria to the nucleus. In order to determine if the p45-FADD interaction interferes with caspase-8 recruitment and activation through the Fas/FADD complex, we generated MCF7 stable cell lines expressing both Fas and p45. As shown in Figure 2B, FasL-induced recruitment of caspase 8 into the DISC is attenuated by p45. Furthermore, the activation of caspase-8 was delayed and the levels of activated caspase-8 were decreased in cells over-expressing p45 (Figure 2C). When the cleavage of PARP-1 was examined, similar results were observed (Figure 2D). These findings were consistently obtained from two independent clones. We then measured whether p45 attenuated cell death induced by Fas activation. Control and p45 over-expressing cells were treated with FasL or an anti-Fas antibody that induces the aggregation of Fas, resulting in the activation of Fas and cell death. As shown in Figure 2E, p45 over-expression markedly reduced cell death induced by both treatments.

### P45 Decreases Injury-induced Cell Death Following Spinal Cord Injury (SCI)

Consistent with increased cell death following SCI [10], expression of Fas and FADD is up-regulated [8], [9]. The ability of p45 to attenuate FasL-induced caspase-8 activation and cell death *in vitro* led us to ask whether p45 inhibits injury-induced cell death *in vivo*. Because p45 expression is dramatically reduced in adulthood (Figure 3A and 3B), we generated several lines of transgenic mice expressing p45 under the control of the Thy1 promoter (Thy1-p45). As shown in Figure 3C and 3D, p45 was highly expressed in the brain and spinal cord of the Thy1-p45 transgenic mice (line #95). Similar results were observed in multiple lines (#96, 99 and 114) of the transgenic mice (data not shown).

**Figure 4:**
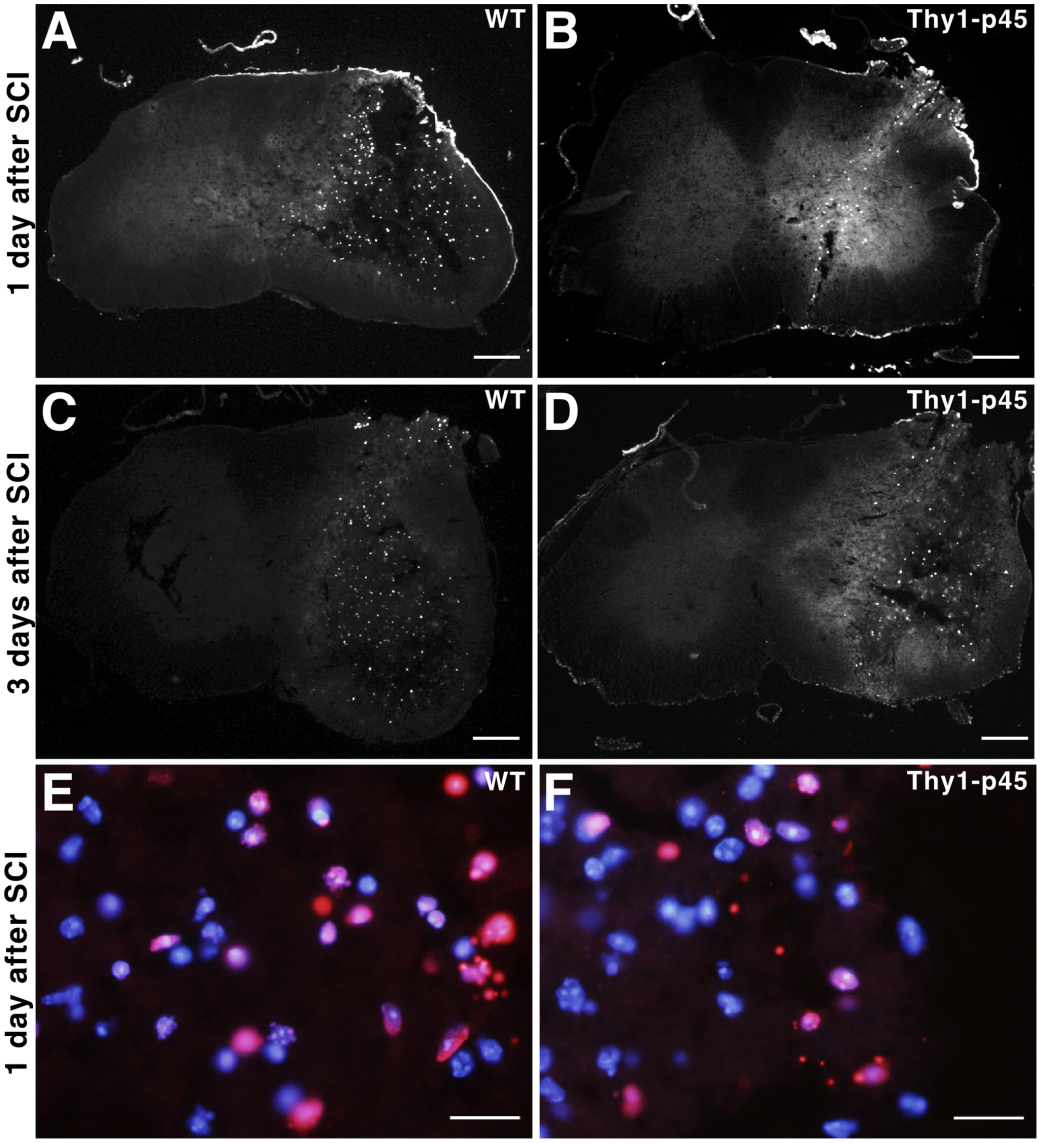
TUNEL assay shows that Thy1-p45 mice have decreased cell death following stab wound injury compared to their WT littermates. One (A, B) or 3 days (C, D) after stab wound injuries on the right side of T13 spinal cord, Thy1-p45 mice (B, D) had fewer TUNEL^+^ cells compared to their WT littermates (A, C) on the injured side of the spinal cord. Serving as an internal control, there were almost no TUNEL^+^ cells on the contralateral uninjured side of the spinal cord in every section. The TUNEL^+^ cells co-localized with Hoechest 33342 nuclei staining and showed the typical features of cell death (DNA fragmentation and condensation) in the magnified view of injured spinal cord from both Thy1-p45 mice and their WT littermates (E–F). Scale bars: 200 µm (A–D), 20 µm (E, F).

**Figure 5:**
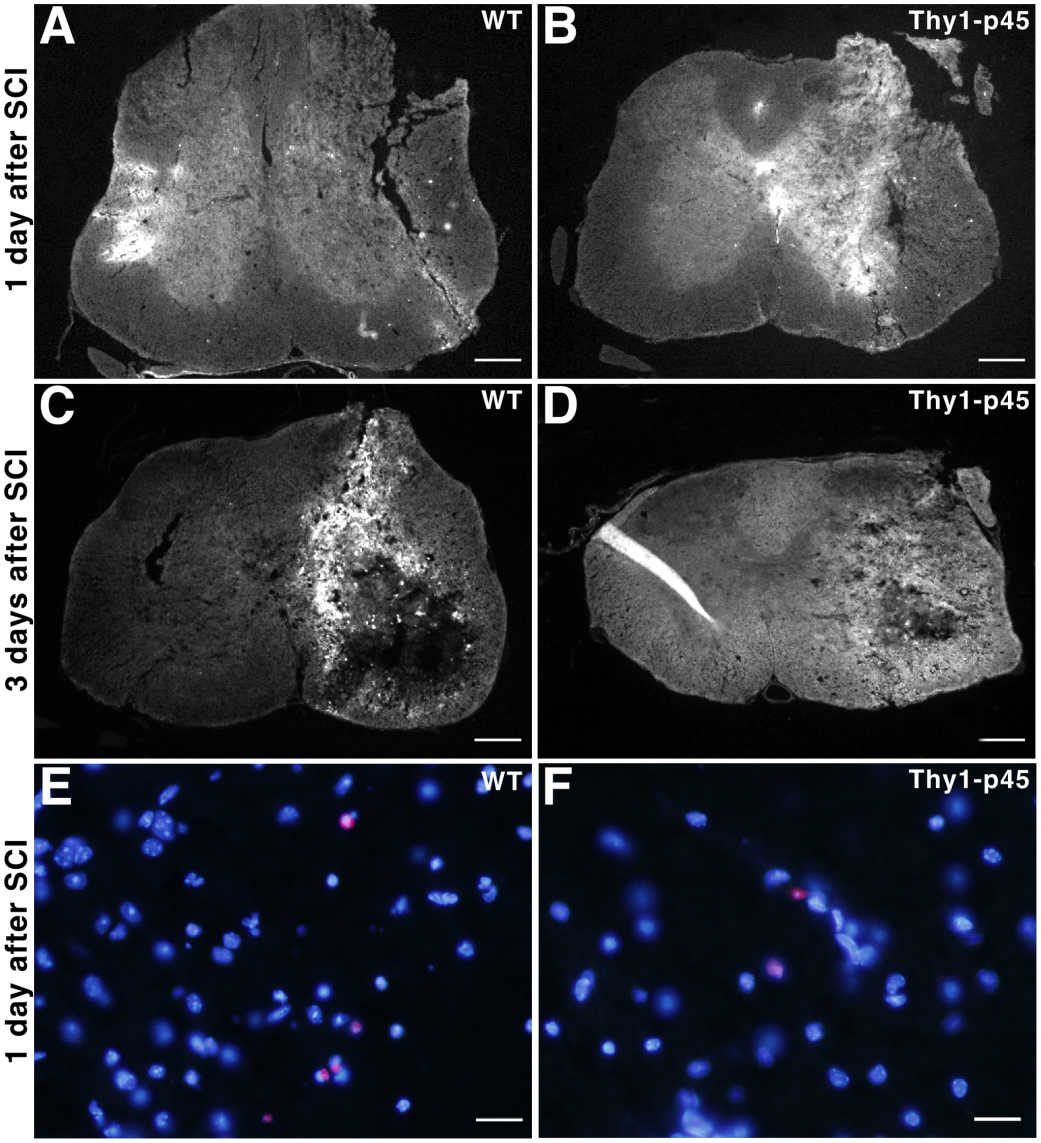
Activated caspase-3 immunohistochemistry shows that Thy1-p45 mice have reduced cell death following stab wound injury compared to their WT littermates. One (A, B) or 3 days (C, D) after stab wound injuries on the right side of T13 spinal cord, Thy1-p45 mice (B, D) had fewer activated caspase-3^+^ cells compared to their WT littermates (A, C) on the injured side of the spinal cord. Serving as an internal control, there were almost no activated caspase-3^+^ cells on the contralateral uninjured side of the spinal cord in every section. The activated caspase-3^+^ cells co-localized with Hoechest 33342 nuclei staining and showed the typical features of cell death (DNA condensation) in the magnified view of injured spinal cord from both Thy1-p45 mice and their WT littermates (E, F). Scale bars: 200 µm (A–D), 20 µm (E, F).

Thy1-p45 transgenic mice do not display an overt phenotype under normal physiological conditions. For example, transgenic mice exhibit normal motor coordination as demonstrated by their standard performance on the Rotarod (data not shown). To determine if p45 exerts pro-survival effects under non-standard physiological conditions, such as after SCI, Thy1-p45 mice were subjected to stab wound injury with a sharp surgical knife as previously described [19] and examined for cell survival. Cell death around the lesion area was measured in several ways. The number of TUNEL^+^ cells was significantly reduced in Thy1-p45 mice at 1 and 3 days following the injury compared to wild type (WT) littermates (Figure 3E and Figure 4). Similar results were observed when the number of cells positive for activated caspase-3 (Figure 3F and Figure 5) was used to measure cell death. Six weeks after injury, we examined the number of cells that were positive for the neuronal marker NeuN (NeuN^+^) 1–2 mm distal from the injury site [30]. As shown in Figure 3G and Figure S1, the number of NeuN^+^ cells within 1 mm distance from the injury site was significantly higher in Thy1-p45 mice compared to WT littermates. Taken together, these results support the finding that p45 over-expression reduces injury-induced cell death in the spinal cord.

### Thy1-p45 Mice Display Increased Functional Recovery Following SCI

**Figure 6:**
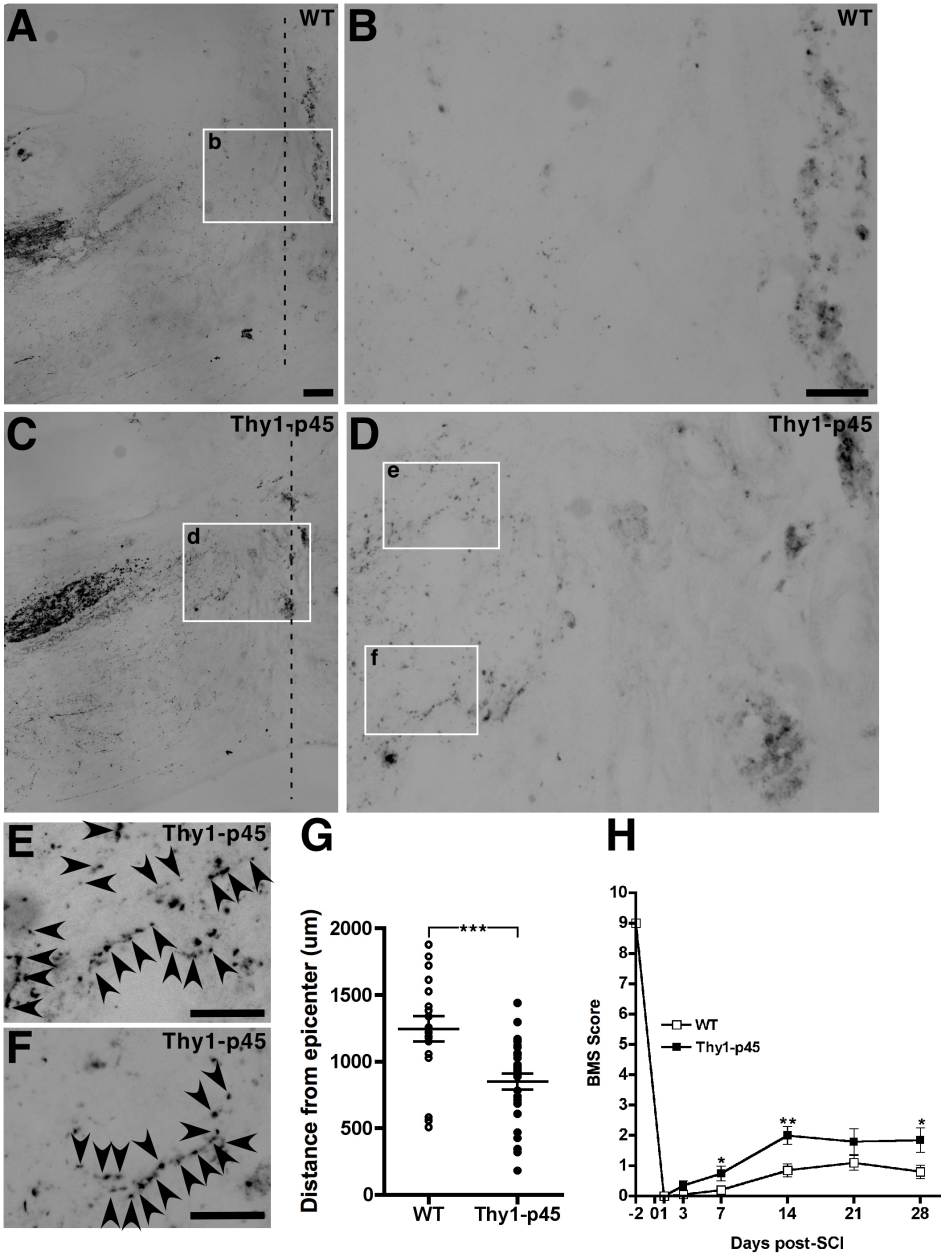
Thy1-p45 transgenic mice have decreased retraction of CST fibers and improved functional recovery following SCI compared to their WT littermates. (A–F) Representative pictures of the YFP-labeled CST fiber front near the lesion epicenter following SCI in the WT littermates (A, B) and Thy1-p45 transgenic mice (C–F). The lesion epicenter is indicated by a vertical dashed line (A and C). (B) A higher magnification of boxed region in (A). (D) A higher magnification of boxed region in (C). (E, F) Higher magnifications of boxed regions in (D). Compared to their WT littermates, the CST fiber front in Thy1-p45 transgenic mice is closer to the lesion epicenter and has increased sprouting (arrows) near the lesion site. Scale bars: 200 µm (A, C), 100 µm (B, D) and 50 µm (E, F). (G) Quantitative analysis of distance between the CST fiber front and the lesion epicenter following SCI. WT littermates, n = 18 (6 mice, 3 sections/mouse), Thy1-p45 transgenic mice, n = 27 (9 mice, 3 sections/mouse). (H) Comparison of the locomotor activity in WT littermates and Thy1-p45 transgenicmice using the BMS scoring system. *P<0.05; **P<0.01; ***P<0.001. Data are represented as means ± s.e.m.

In order to determine if the survival-promoting effects of p45 over-expression observed after injury are correlated with enhanced behavioural performance, we performed spinal cord transections in WT and p45 over-expressing mice and analysed functional recovery. Thirteen-week-old Thy1-p45 and WT littermates were subjected to spinal cord transection at thoracic level 9 (T9). We used the Basso Mouse Scale (BMS) to quantitatively assess functional recovery of SCI [20]. On day 1 after SCI, all mice had a BMS score of 0, indicating they were completely paraplegic. With time, Thy1-p45 transgenic mice exhibited weight-bearing postures and non-plantar stepping. As shown in Figure 6H, locomotor performance following each time point was significantly better in Thy1-p45 transgenic mice compared to their WT littermates. These results demonstrate that over-expression of p45 promotes functional recovery following SCI.

Next, we sought to determine whether the improved functional recovery is attributed to decreased degeneration and/or increased re-growth of descending motor pathways that had been severed in the Thy1-p45 mice. To assess the growth of corticospinal tract (CST), the CST fibers were either anterogradely labeled with biotin-dextran amine (BDA) injection into the sensory-motor cortex of injured mice 4 weeks post-SCI or genetically labeled with YFP using Emx1-Cre/Thy1-STOP-YFP mice as previously described [31]. Similar results were observed. There was significantly less retraction of CST fibers from the epicenter of injury (Figure 6G and Figure S2; WT, 1244±94.82 µm, n = 18; Thy1-p45, 849.0±59.81 µm, n = 27; p<0.001) and a higher number of collateral CST sprouts in Thy1-p45 mice compared to WT littermates (Figure 6A–6F). As shown in Figure 6C and 6D, there were YFP-labeled nerve fibers close to the lesion site in Thy1-p45 transgenic mice, but not in their WT littermates (Figure 6A and 6B). Higher magnification of the CST fibers close to the lesion site in Thy1-p45 mice showed that they had a morphology resembling synaptic boutons (Figure 6E and 6F). These results suggest that the increased functional recovery in Thy1-p45 transgenic mice may in part be due to decreased retraction and increased sprouting of severed CST tracts. Interestingly, decreased retraction is also observed following injury in mice either treated with FasL-specific neutralizing antibodies [6] or deficient in a receptor for chondroitin sulfate proteoglycan, an inhibitor of neural regeneration [22], [32].

## Discussion

P45 is a unique member of the DD superfamily in that, from its amino acid sequence, its intracellular domain has the highest homology with p75 and considered as a DD. However, the DD of p45 contains a specific RxDφ motif at its α6-helix that is important for holding DED structure. Therefore, it may possess certain binding characteristics of DED. Our studies showed that p45 is able to bind FADD. FADD is composed of a DD and a DED. Deletion constructs and amino acid mutation constructs of FADD suggest that the DED of FADD is necessary for its binding with p45. FADD mediates FasL-induced apoptotic signals by binding to Fas through its DD and recruits pro-caspase-8 through its DED, thus forming the DISC to transduce apoptotic signals. Since the DED domain is critical for the interaction of FADD with p45, and amino acid mutations that prevent pro-caspase-8 binding with FADD also abolish the interaction between FADD and p45, it is conceivable that p45 and pro-caspase-8 share the same binding surface in the DED of FADD. Therefore, over-expression of p45 may competitively block the recruitment of pro-caspase-8 by FADD and reduce the pro-apoptotic signals induced by FasL. An alternative explanation of these results is that the FADD –FADD dimerization and/or FADD-caspase-8 complex formation are crucial for p45-FADD interaction. Whether p45 executes its biological functions by only interacting with FADD-FADD and/or FADD-caspase-8 complex needs further studies.

Before the results of our studies, it was thought that most interactions between DD superfamily members are homotypic, meaning only domains from the same subfamily bind to each other. However, p45 can bind both proteins containing a DD, such as p75 (Vilar *et al.,* submitted), and DED-containing proteins, such as FADD. By interacting with p75 through its DD, p45 is able to reduce p75/Nogo receptor-mediated nerve growth inhibition from myelin-associated inhibitors and may also contribute to functional recovery in the Thy1-p45 mice following injury. These results greatly expand the functionality and signaling pathways influenced by p45. Furthermore, the p45 ICD has been implicated in binding to intracellular domains that do not have the features of DD or DED domain. For example, p45 ICD is able to interact with the intracellular domain of the TrkA receptor and regulate TrkA-mediated Erk1/2 activation [17]. The ability to interact with non-DD superfamily molecules is also found in other DD superfamily members, including PEA-15 [23], [33], [34], [35], [36]. The versatility of p45’s interactions makes it a switch that can influence multiple signaling pathways.

The functional recovery after SCI involves multiple biological processes such as cell survival, nerve growth, myelination and synapse formation [37]. After SCI, FasL, Fas, and FADD are up-regulated in the local environment around the injury site and have been shown to play a role in promoting cell death and preventing functional recovery [6]. In addition to FasL, many other factors in the local environment can influence the outcome of functional recovery, such as growth factors [38], chondroitin sulfate proteoglycans [39] and myelin-associated inhibitors [40]. One major challenge in treating SCI is to develop strategies that can affect multiple signaling pathways simultaneously and synergistically [41]. The multitude of concerted regulations by p45 makes it a powerful target for developing treatments for SCI. Understanding p45-mediated cellular and molecular mechanisms may provide insights into facilitating nerve regeneration in humans.

## Supporting Information

Figure S1 **Thy1-p45 mice have enhanced neuronal survival in regions located close to, but not at the epicenter of stabbing wound injury compared to their WT littermates at 6 weeks after injury.** Six weeks after stabbing wound injuries on the right side of T13 spinal cord, Thy1-p45 mice (B, D, F, H) and their WT littermates (A, C, E, G) were analyzed for neuronal survival using immnohistochemistry with a NeuN-specific antibody. Serving as an internal control, there were NeuN+ cells in the grey matter on the uninjured side of the spinal cord in every section. Six weeks after the stabbing wound injury, there were more NeuN^+^ cells in regions 0–1 mm posterior from the epicenter of the injury in Thy1-p45 mice (F) compared to their WT littermates (E). There was no significant difference in the number of NeuN^+^ cells between Thy1-p45 mice (B, D) and WT littermates (A, C) in regions at the epicenter of the injury (A–D). There was also no significant difference in the number of NeuN^+^ cells between Thy1-p45 mice (G) and WT littermates (H) in regions 1–2 mm posterior from the epicenter of the injury. Scale bar, 200 µm.
(TIF)

Figure S2 **Quantification CST fiber retraction: p45 over-expression decreases retraction of the CST fibers following SCI.** Thy1-p45 transgenic mice and their WT littermates received SCI, and CST fibers were labeled by either axon tracing with BDA or genetic YFP labeling as described in methods. Representative pictures of the CST fibers front near the lesion epicenter at 6 weeks post-SCI in the WT littermates (A) and Thy1-p45 transgenic mice (B) are shown. The lesion epicenter defined as the middle point of the scar on each sagittal spinal cord section is indicated by a vertical dashed line (A, B). Similar to the CST fiber retraction analysis described previously [22], the distance (horizontal white lines, A, B) between the lesion epicenter and the main CST fiber front, which was defined as the point where adjacent fibers form a fascicle that is 100 µm (A, B) wide or more was measured. Three qualified sections from each mouse were analyzed. Scale bars: 200 mm (A, B). (C) Quantitative analysis of the distance between the CST fiber front and the lesion epicenter following SCI. WT littermates, 1244±94.82 µm, n = 18 (6 mice, 3 sections/mouse); Thy1-p45 transgenic mice, 849.0±59.81 µm, n = 27 (9 mice, 3 sections/mouse). ***p<0.001.
(TIF)
