# Supplementary figures and images for: P45 Forms a Complex with FADD and Promotes Neuronal Cell Survival Following Spinal Cord Injury
Source: PLoS One. 2013 Jul 23;8(7):e69286. doi: 10.1371/journal.pone.0069286 (PMC3720591; doi:10.1371/journal.pone.0069286)

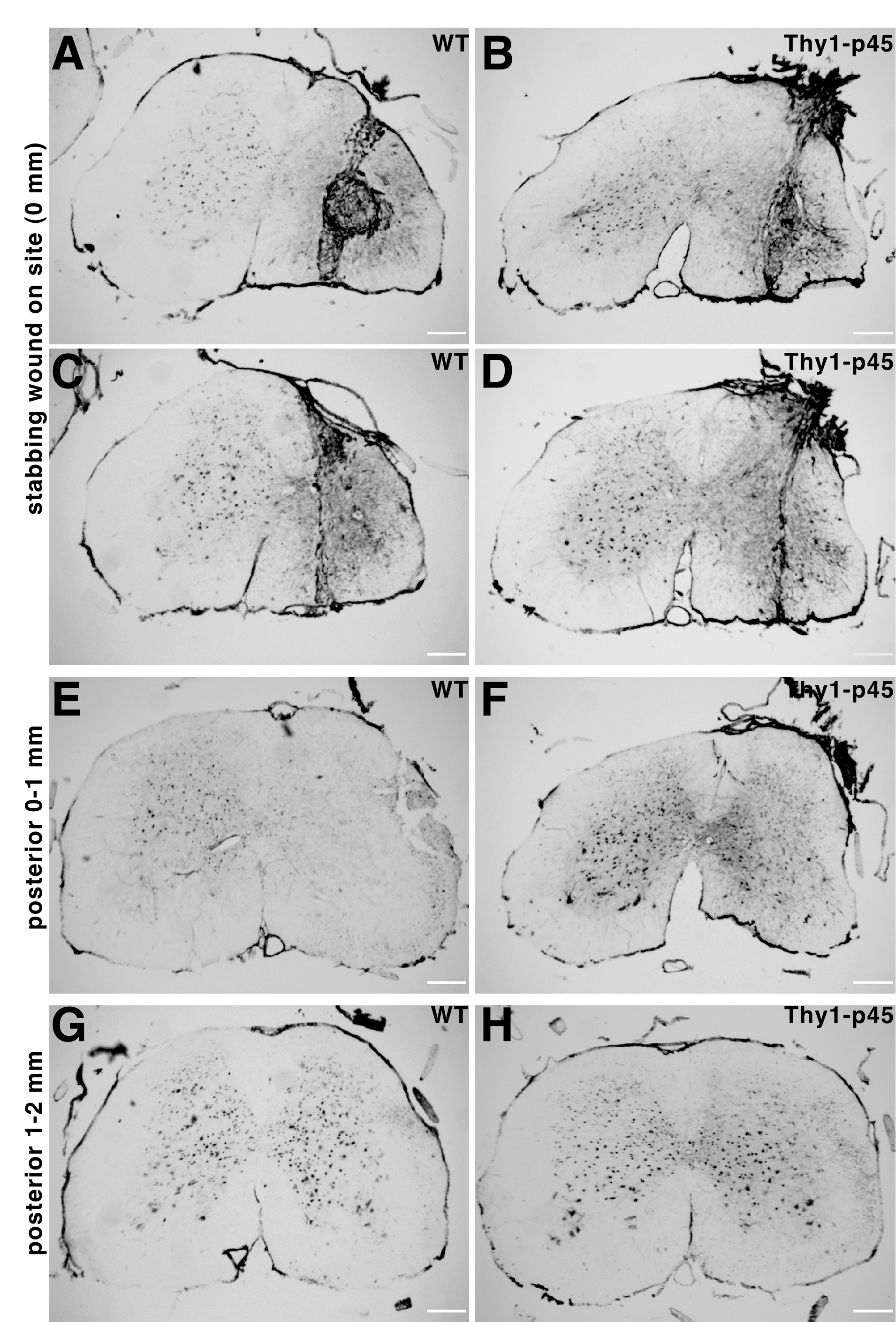

Supplement: Figure S1 — Thy1-p45 mice have enhanced neuronal survival in regions located close to, but not at the epicenter of stabbing wound injury compared to their WT littermates at 6 weeks after injury. Six weeks after stabbing wound injuries on the right side of T13 spinal cord, Thy1-p45 mice (B, D, F, H) and their WT littermates (A, C, E, G) were analyzed for neuronal survival using immnohistochemistry with a NeuN-specific antibody. Serving as an internal control, there were NeuN+ cells in the grey matter on the uninjured side of the spinal cord in every section. Six weeks after the stabbing wound injury, there were more NeuN+ cells in regions 0–1 mm posterior from the epicenter of the injury in Thy1-p45 mice (F) compared to their WT littermates (E). There was no significant difference in the number of NeuN+ cells between Thy1-p45 mice (B, D) and WT littermates (A, C) in regions at the epicenter of the injury (A–D). There was also no significant difference in the number of NeuN+ cells between Thy1-p45 mice (G) and WT littermates (H) in regions 1–2 mm posterior from the epicenter of the injury. Scale bar, 200 µm. (TIF) [file pone.0069286.s001.tif]

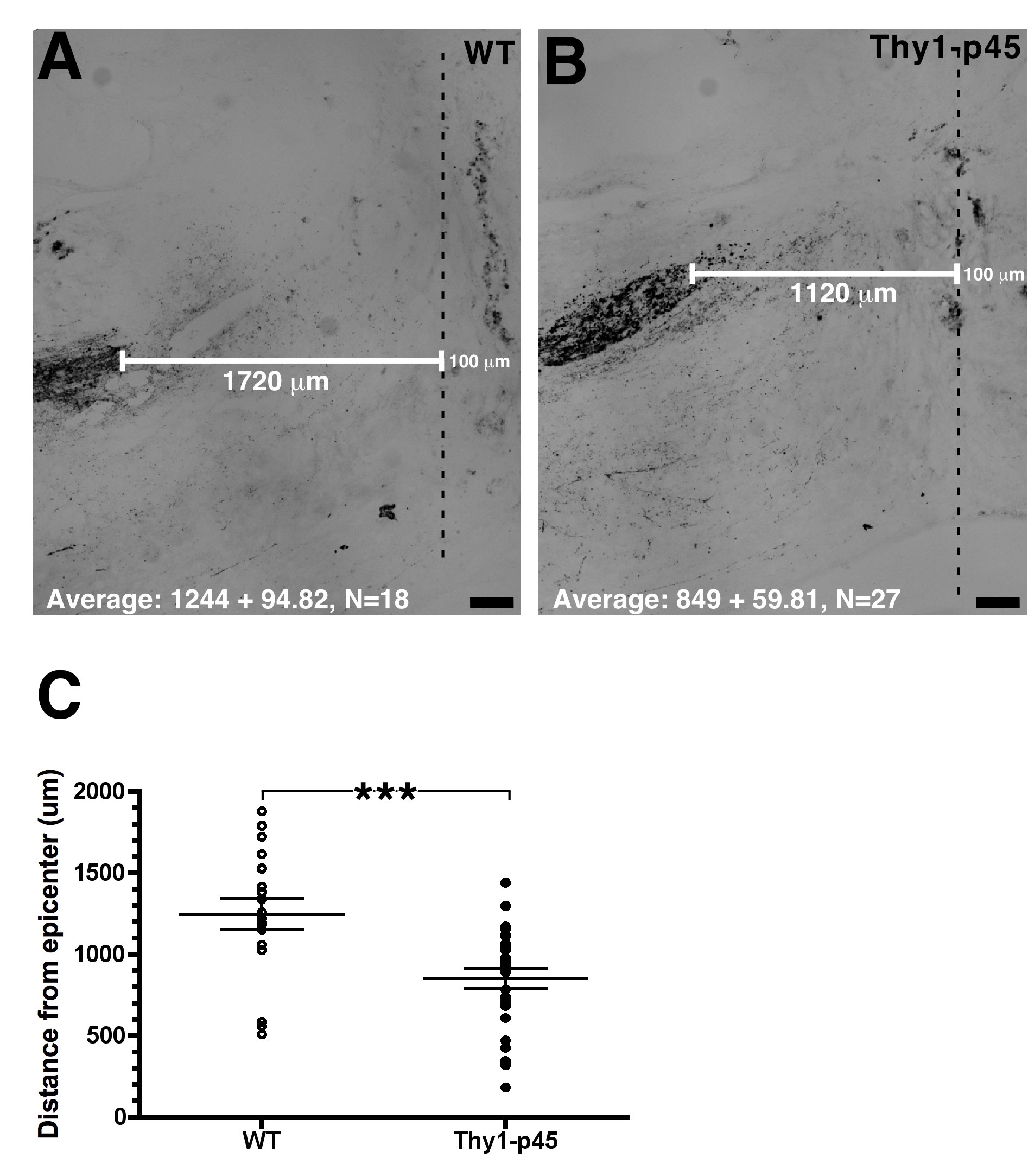

Supplement: Figure S2 — Quantification CST fiber retraction: p45 over-expression decreases retraction of the CST fibers following SCI. Thy1-p45 transgenic mice and their WT littermates received SCI, and CST fibers were labeled by either axon tracing with BDA or genetic YFP labeling as described in methods. Representative pictures of the CST fibers front near the lesion epicenter at 6 weeks post-SCI in the WT littermates (A) and Thy1-p45 transgenic mice (B) are shown. The lesion epicenter defined as the middle point of the scar on each sagittal spinal cord section is indicated by a vertical dashed line (A, B). Similar to the CST fiber retraction analysis described previously [22], the distance (horizontal white lines, A, B) between the lesion epicenter and the main CST fiber front, which was defined as the point where adjacent fibers form a fascicle that is 100 µm (A, B) wide or more was measured. Three qualified sections from each mouse were analyzed. Scale bars: 200 mm (A, B). (C) Quantitative analysis of the distance between the CST fiber front and the lesion epicenter following SCI. WT littermates, 1244±94.82 µm, n = 18 (6 mice, 3 sections/mouse); Thy1-p45 transgenic mice, 849.0±59.81 µm, n = 27 (9 mice, 3 sections/mouse). ***p<0.001. (TIF) [file pone.0069286.s002.tif]
